# Health systems strengthening, universal health coverage, health security and resilience

**DOI:** 10.2471/BLT.15.165050

**Published:** 2016-01-01

**Authors:** Joseph Kutzin, Susan P Sparkes

**Affiliations:** a World Health Organization, avenue Appia 20, 1211 Geneva 27, Switzerland.

Global and national initiatives focused on health systems strengthening, universal health coverage, health security, and resilience suffer when these terms are not well understood or believed to be different ways of saying the same thing. Here we aim to facilitate understanding and highlight key policy considerations by identifying critical attributes of each concept and emphasizing the distinction between ends and means in health policy.

Set within the political and institutional framework of a country, a health system is “the ensemble of all public and private organizations, institutions, and resources mandated to improve, maintain or restore health.”^1^ This definition, along with efforts to more concretely specify the “functions”, “building blocks”, or “control knobs” of a health system, focus on the characteristics or policy instruments of the system itself.^2^^–^^4^ Strengthening health systems involves “a significant, purposeful effort to improve performance.”^4^ This goes beyond merely investing in inputs; it means reforming how the health system actually operates.^5^

Universal health coverage means that all people are able to receive needed health services of sufficient quality to be effective, without fear that the use of those services would expose the user to financial hardship.^6^ Based on this definition, universal health coverage comprises a set of objectives – equity in service use, quality, and financial protection – towards which all countries strive. Progress is assessed at population scale, rather than only those served by specific schemes or programmes.^7^ Non-discrimination is a core principle; policies that exclude certain individuals or groups are inconsistent with universal health coverage.^8^ Because people need individual and public health services, ensuring that both are delivered effectively falls within the scope of universal health coverage. Criticizing the universal health coverage concept by arguing that public health services are excluded^9^ is wrong, though in practice this argument may have validity. Finally, universal health coverage is globally relevant; all countries can do something to reduce the gap between the need for and the use of quality health services.^6^

Health system strengthening comprises the means (the policy instruments), while universal health coverage is a way of framing the objectives of policy. Without this distinction, there is a risk that instruments of reform become the objective, with the perception that “the problem” to be solved is the absence or presence of a particular policy instrument. When this occurs, policy dialogue shifts quickly away from where it needs to be – getting to consensus about the nature and causes of underperformance relative to universal health coverage goals – to what is often an ideologically polarized debate about the inherent merits or flaws of particular reform instruments. In health financing, for example, this has been observed in the debate on social or community-based health insurance, performance-based financing and user fees. Similarly, simply calling something a “universal health coverage reform” does not convey any meaning as to the actual content of what is being planned or implemented.

Beyond the objectives embedded in universal health coverage, it entails individual health security^10^ the intrinsic value of protection against risk.^11^ Individuals are better off when they are secure in the knowledge that if something should happen they will be able to obtain quality health services without becoming impoverished as a result. Collective health security^10^ – reducing the vulnerability of societies to health threats that spread across national borders – is a goal that extends beyond the definition of universal health coverage.  But there is a clear link, because health systems that progress towards universal health coverage also contribute to collective health security. Therefore, health systems strengthening is needed to make progress towards universal health coverage and health security.

The resilience of a health system refers to its ability to absorb disturbance, to adapt and respond with the provision of needed services.^12^ Thus, resilience is not an action to be implemented but rather a dynamic objective of investments and reforms. In the case of Ebola-affected countries, for example, this has required efforts to not only restore how the system functioned before the crisis but to transform and fundamentally improve the health system.

Conceptual clarity is essential for a systematic approach to policy-making. Confusion and inefficiency arise when health system strengthening is defined as an objective and also when universal health coverage, health security or resilience are described as separate programmes to be implemented. So here is a simple guide: health system strengthening is what we do; universal health coverage, health security and resilience are what we want.
